# Organic and biochar-based fertilizers reshape bacterial communities and enhance fertility in acidic rubber plantation soils

**DOI:** 10.3389/fmicb.2026.1861571

**Published:** 2026-07-14

**Authors:** Jifen Yang, Xiaoling Shi, Shunjun Geng, Chunxia Yang, Muguo Xu

**Affiliations:** 1 Yunnan Key Laboratory of Sustainable Utilization Research on Rubber Tree, Jinghong, Yunnan, China; 2 Yunnan Institute of Tropical Crops, Jinghong, Yunnan, China

**Keywords:** acidic rubber plantation soils, bacterial community structure, fertilization, *Hevea brasiliensis*, soil restoration

## Abstract

**Introduction:**

Understanding the effects of different soil amendments on bacterial community structure and diversity in acidic rubber plantation soils is essential for biological remediation and the targeted restoration of these degraded systems.

**Methods:**

In this study, a 2.5-year long field experiment was conducted in a second-generation 25-year-old rubber plantation at Dongfeng Farm in Jinghong City, southern Yunan, China. The experiment included five fertilization treatments: (1) unfertilized control (CK), (2) microbial fertilizer (T1), (3) biochar-based fertilizer (T2), (4) tobacco ash and oil cake organic fertilizer (T3), and (5) bio-organic fertilizer combined with polyacrylamide (T4). Soil samples were collected from the 0–20 cm layer in October 2022, and bacterial communities were analyzed using Illumina MiSeq high-throughput sequencing. Key environmental drivers were identified by integrating sequencing data with soil physicochemical properties.

**Results:**

The application of soil fertilizers significantly altered the tested soil physicochemical properties and bacterial community composition. Compared with CK, T1 and T4 increased soil pH. T2 and T3 significantly enhanced soil organic matter, available phosphorus, and total nitrogen. In addition, T2 specifically increased exchangeable Ca^2+^ and Mg^2+^ concentrations and elevated the Chao1 richness index. Both T2 and T3 enriched beneficial taxa, including Proteobacteria and *BradyRhizobium*, while principal coordinate analysis (PCoA) revealed distinct shifts in bacterial community structure. The T4 treatment resulted in the most complex bacterial co-occurrence network. Mantel tests identified organic matter, total nitrogen, available phosphorus, and available nitrogen as the primary drivers of bacterial diversity. Further analysis using structural equation modeling indicates that soil conditioners alter bacterial community structure and diversity by influencing soil fertility and pH.

**Discussion:**

Collectively, these findings demonstrate that organic matter, total nitrogen, available phosphorus, and available nitrogen serve as key environmental factors shaping bacterial community structure and diversity in acidic rubber plantation soils. Organic fertilizers and biochar have proven highly effective in enhancing soil fertility and buffering capacity, while significantly increasing the abundance of dominant bacterial taxa. Therefore, organic and biochar-based amendments should be prioritized as effective and sustainable strategies for restoring soil health and promoting continuous soil quality improvement in acidified rubber plantations.

## 1 Introduction

*Hevea brasiliensis* is an economically significant tree species cultivated in tropical regions of China, playing a vital role in securing strategic national material supplies and promoting regional economic development. However, decades of intensive cultivation have led to substantial soil degradation in rubber plantations, with soil acidification posing a particularly critical threat to long-term productivity and ecosystem sustainability (Sun R. et al., 2025). The continuous application of nitrogen fertilizers, combined with the secretion of organic acids from rubber tree roots and the release of acidic compounds during litter decomposition, collectively contributes to a marked decline in soil pH in these systems (Tian and Niu, 2015; Fujii et al., 2024). Soil acidification in rubber plantations mobilizes phytotoxic aluminum and manganese ions, which severely impair root growth and function while reducing the availability of essential nutrients (Jessica et al., 2006; Chen and Liao, 2016; Jiayi, 2023). A study conducted in Xishuangbanna revealed that the contents of exchangeable calcium, magnesium, available zinc, manganese, and iron in rubber plantation soils were positively correlated with pH, whereas exchangeable aluminum content showed a negative correlation (Liu et al., 2019). Furthermore, excessively high aluminum concentrations have been shown to induce damage to rubber tree cell membranes, thereby inhibiting growth (Ma et al., 2021; Ling et al., 2023). Collectively, these findings underscore the necessity of developing effective strategies to increase soil pH and mitigate acidification to ensure the sustainable growth and productivity of rubber plantations.

Soil microorganisms, particularly bacteria, represent the most active and abundant biotic components in soil ecosystems and play a pivotal role in nutrient cycling and organic matter decomposition (Liao et al., 2018; Dong et al., 2026). As primary drivers of biogeochemical cycles, they are fundamental to soil formation, litter decomposition, and nutrient transformation processes, and serve as key regulators of soil nutrient supply capacity (Chang et al., 2023). Soil microbial diversity and community composition are strongly correlated with soil pH across a wide range of spatial scales (Duan et al., 2025). As a key determinant of microbial diversity (Sun et al., 2015), a decline in soil pH suppresses the metabolic activity and proliferation of acid-sensitive bacterial groups, leading to reduced community diversity and impaired soil nutrient supply (Liu et al., 2018). Therefore, elucidating the response mechanisms of bacterial communities in acidified rubber plantation soils to various amelioration practices is essential for understanding the microecological processes underlying soil degradation and recovery.

The application of soil amendments is widely recognized as an effective strategy for mitigating soil acidification and restoring soil health. Appropriate amendment application of soil amendments can improve soil physicochemical properties, revitalize microbial activity, and suppress soil-borne diseases (Qiang, 2024). By regulating the soil microenvironment, soil amendments influence microbial activity and drive shifts in microbial community structure (Lei et al., 2024; Zhang et al., 2025). Currently, amendments such as biochar, organic fertilizers, and lime are commonly employed for acidic soil remediation. Organic fertilizers improve soil buffering capacity, enhance fertility, and provide available carbon (C) sources, thereby promoting microbial proliferation (Zhen et al., 2019). Biochar creates favorable microhabitats for soil microorganisms by increasing soil pH, porosity, and water and nutrient retention capacity (Shiv et al., 2023). Lime application indirectly affects microbial community structure primarily by alleviating soil acidification and increasing the supply of available nutrients such as calcium and magnesium (Shengnan et al., 2021). Despite these insights, previous studies have largely focused on the short-term effects of soil chemical properties on crop yield, leaving a gap in the systematic understanding of the long-term causal pathways linking different soil amendments to changes in the soil microenvironment, microbial community structure, and network stability. Moreover, empirical evidence based on high-throughput sequencing-derived co-occurrence networks remains particularly limited.

To address this knowledge gap, this study investigated typical acidified rubber plantations in Xishuangbanna, Yunnan Province, China. Using high-throughput 16S rRNA gene Illumina MiSeq sequencing, combined with analyses of soil microbial co-occurrence networks and community composition, we systematically elucidated the mechanisms by which different soil conditioners influence bacterial community structure and diversity in rubber plantation soils. Specifically, this study aimed to answer the following questions: (1) What are the regulatory mechanisms through which different soil conditioners modulate bacterial community structure and diversity in rubber plantations under long-term application? (2) How do different conditioners affect the stability of microbial co-occurrence networks? And (3) What are the key environmental drivers shaping bacterial community structure and diversity? The findings provide a theoretical foundation for targeted microbial regulation and precision remediation of acidified soils in rubber plantations. Furthermore, this research has important implications for reducing chemical fertilizer use, promoting green and low-C production practices, and safeguarding the national strategic security of natural rubber resources.

## 2 Materials and methods

### 2.1 Study site overview and experimental design

A field experiment was conducted from March 2020 to October 2022 at Sub-field 6 of Dongfeng Farm, Jinghong City, Yunnan, China (21°58′37″ N, 100°65′78″E, elevation 683 m). The site has a tropical monsoon climate, with an annual mean temperature of 23 °C and annual mean precipitation of 1,400 mm. The rubber tree *Hevea brasiliensis* cv. RRIM600 was planted in 1998 and tapped since 2005 with a spacing of 2.5 m × 9.0 m. The initial physicochemical properties of the topsoil layer (0–20 cm) were as follows: pH 4.12, total N (TN) 1.32 g·kg^−1^, soil organic matter (SOM) 28.25 g·kg^−1^, alkali-hydrolyzable nitrogen (AN) 117.07 mg·kg^−1^, available phosphorus (AP) 1.92 mg·kg^−1^, and available potassium (AK) 22.17 mg·kg^−1^.

The experiment employed a single-factor randomized block design with five treatments, each replicated across three plots, for a total of 15 plots. Each plot measured 10 m × 30 m (300 m^2^), with a minimum separation distance of 20 m between adjacent plots. The five treatments were as follows: (1) unfertilized control (CK), (2) microbial fertilizer (T1), (3) biochar-based fertilizer (T2), (4) tobacco ash and oil cake organic fertilizer (T3), and (5) bio-organic fertilizer combined with polyacrylamide (T4). The microbial fertilizer (Huafeng Technology Co., Ltd., Linyi, Shandong, China) contained *Bacillus subtilis* at a concentration of ≥ 3.0 × 10^8^ CFU·g^−1^ and had an SOM content of ≥ 45%. The biochar-based fertilizer (Guangdong Gaya Ecological Co., Ltd., Guangzhou, Guangdong, China) had an organic matter content of ≥ 60% and a humic acid content of ≥ 25%. The bio-organic fertilizer (Tonghai Modern Bio-fertilizer Co., Ltd., Tonghai, Yunnan, China) had an organic matter content ≥ 40% and contained ≥ 0.2 billion CFU·g^−1^ of effective viable bacteria (*Bacillus subtilis*, *Bacillus licheniformis*, and *Bacillus amyloliquefaciens*). The tobacco ash and oil cake organic fertilizer (Tonghai Modern Bio-fertilizer Co., Ltd., Tonghai, Yunnan, China) had a SOM content of ≥ 45%. The polyacrylamide was sourced from Sinopharm Group. All fertilizers, except polyacrylamide, were applied at a rate of 2,250 kg·hm^−2^ (equivalent to 5 kg per plant) based on local fertilization standards. Polyacrylamide was applied at 22.5 kg·hm^−2^ according to Zhao et al. (2023). In treatment T4, bio-organic fertilizer and polyacrylamide were co-applied. Fertilizer application trenches (100 cm long, 40 cm wide, and 40 cm deep) were prepared between two adjacent rubber trees along the inner edge of the terraces. All amendments were applied as a single annual application in March 2020, 2021, and 2022.

### 2.2 Sample collection and preservation

After 2.5 years of fertilization, soil sampling was conducted in October 2022. In each plot, soil surrounding the root system adjacent to the fertilization trenches was collected. Rhizosphere soil was defined as the soil firmly attached to root surfaces, which was obtained by gently shaking and carefully detaching the adhering soil from the roots. A portion of the rhizosphere soil was homogenized, passed through a 2-mm sieve, and air-dried for physicochemical property analysis of physicochemical properties. The remaining portion was immediately stored at −80 °C for subsequent DNA extraction.

### 2.3 Methods and analyses

#### 2.3.1 Soil physicochemical properties

All tested soil physicochemical properties including pH, available potassium (AK), available nitrogen (AN), available phosphorus (AP), cation exchange capacity (CEC), exchangeable calcium (ECa), exchangeable magnesium (EMg), organic matter (OM), total nitrogen (TN), and total exchangeable base (TEB) were analyzed using previously described methods (Serafim et al., 2023).

#### 2.3.2 Soil bacterial DNA extraction, PCR amplification, and sequencing

Total soil DNA was extracted from 0.5 g of each sample using the E.Z.N.A.^®^ Soil DNA Kit (Omega Bio-tek, Norcross, GA, USA) following the manufacturer’s instructions. DNA quality was assessed by electrophoresis on 1% (w/v) agarose gel, and DNA concentration and purity were measured using a NanoDrop 2000 spectrophotometer (Thermo Scientific, Waltham, MA, USA). The V3-V4 hypervariable region of the bacterial 16S rRNA gene was amplified using the universal primers 338F (5′-ACTCCTACGGGAGGCAGCAG-3′) and 806R (5′-GGACTACHVGGGTWTCTAAT-3′).

The PCR products were extracted from 2% agarose gels using a DNA gel purification kit. Library preparation utilized the NEXTFLEX Rapid DNA-Seq Kit, with sequencing carried out on the Miseq platform at Shanghai Meiji Biotechnology Co., Ltd. in Shanghai, China. Raw sequencing reads were assembled, quality-filtered, and chimeras removed. Operational taxonomic units (OTUs) were classified at 97% similarity using UPARSE version 7.1. The RDP classifier was employed for taxonomic assignment against the Silva 16S rRNA gene database (version 138), maintaining a 70% confidence threshold. To account for uneven sequencing depth, OTU abundance tables were normalized based on the minimum sample sequence count prior to downstream analyses.

### 2.4 Data analysis

Statistical analyses were performed using SPSS 21.0 (IBM Corp., Armonk, NY, USA). One-way analysis of variance (ANOVA) was followed by Duncan’s multiple range test (*p <* 0.05) was used to evaluate the effects of different treatments on soil physicochemical properties. Before performing one-way ANOVA, we conducted the Shapiro–Wilk normality test and Levene’s homogeneity of variance test on all data. All data met the requirements of normality and homogeneity of variance (*p >* 0.05). With the help of Origin 2021 (OriginLab Corp., Northampton, MA, USA), graphs were generated. Principal coordinate analysis (PCoA) based on Bray-Curtis distances at the OTU level was used to visualize differences in bacterial community composition among treatments. Permutational multivariate analysis of variance (PERMANOVA) was used to test the significance of treatment effects on bacterial community structure. The connections between bacterial communities and environmental factors were visualized through a heatmap. Microbial co-occurrence networks were constructed using R version 4.5.1 (R Core Team, Vienna, Austria), with the “vegan” and “WGCNA” packages used to calculate edge and node properties. Network visualization was subsequently performed using Gephi (version 0.10.1). Structural equation modeling (SEM) was conducted using R version 4.5.1, with the “brms” package used to examine the hypothesized pathways through which soil amendments influence bacterial community structure and diversity.

## 3 Results and analysis

### 3.1 Effects of different amendments on soil physicochemical properties

**Figure 1:**
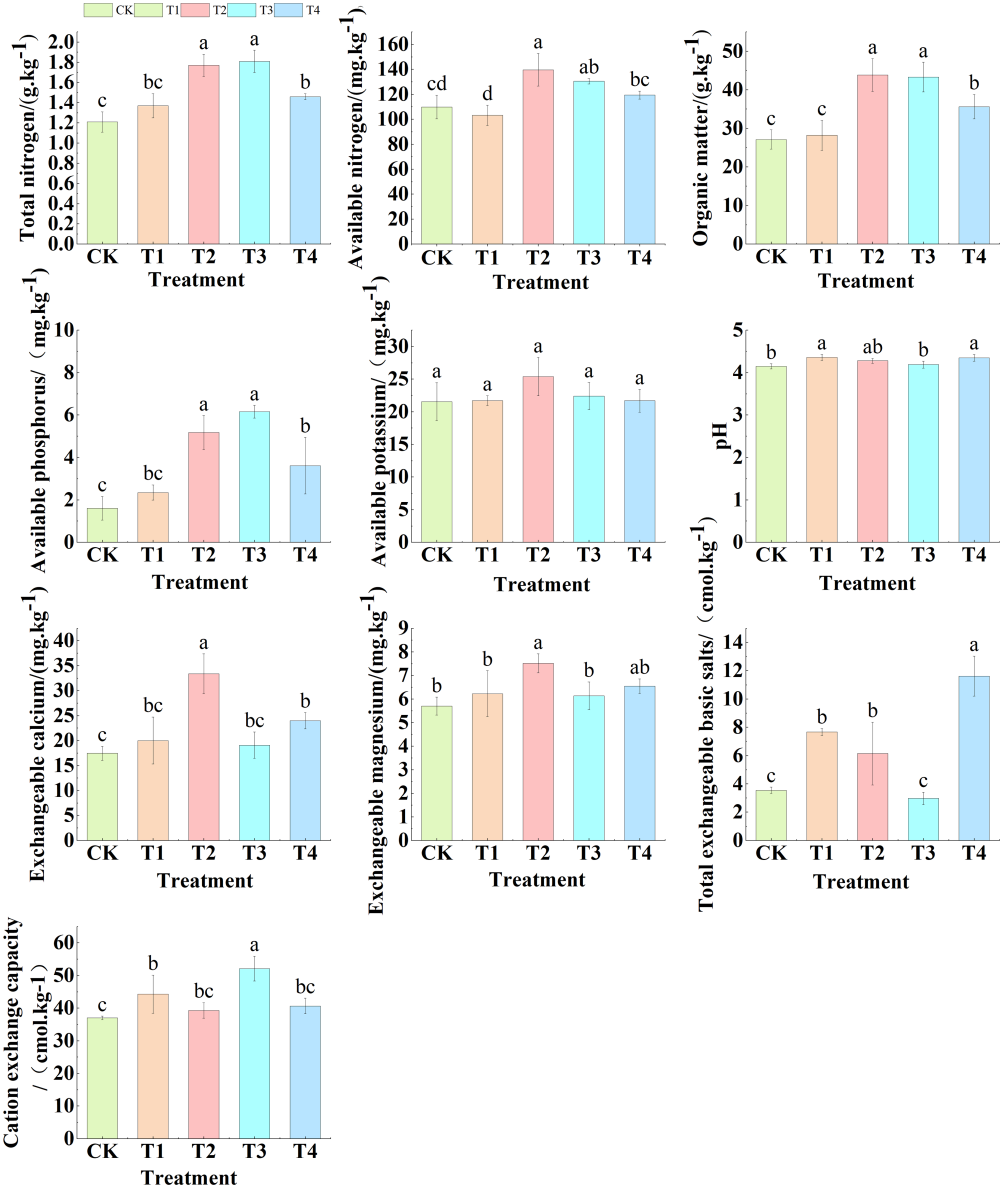
Variation in soil physicochemical properties under different soil conditioners.

The application of soil amendments significantly altered several soil physicochemical properties relative to the control (CK), as summarized in Figure 1. Soil organic matter (SOM) content increased significantly under T2 and T3 compared with CK. Although soil AK content did not differ significantly among treatments (*p <* 0.05), AP content varied significantly (*p <* 0.05), with T2 and T3 showing markedly higher levels than CK, T1, and T4. AN content was significantly higher in T2 than in CK, T1, T3, and T4. Total N content was also significantly elevated under T2 and T3 relative to CK (*p <* 0.05).

Soil pH was significantly higher in T1 and T4 compared with CK. Exchangeable calcium and magnesium contents varied significantly among treatments; exchangeable calcium was significantly greater in T2 than in CK, T3, and T4. Total exchangeable base content was significantly higher in T4 than in CK, T1, T2, and T3 (*p <* 0.05). Additionally, cation exchange capacity (CEC) differed significantly across treatments, with T3 exhibiting significantly higher CEC than CK, T1, T2, and T4 (*p <* 0.05).

### 3.2 Effects of different amendments on soil bacterial alpha diversity

**Figure 2:**
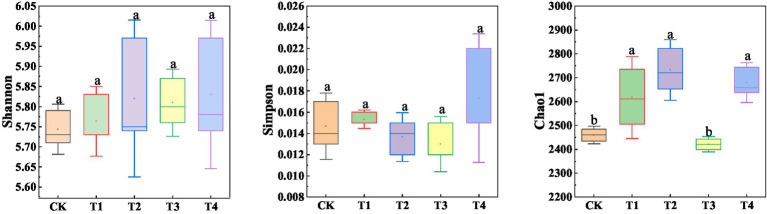
Variation in bacterial *α*-diversity under different soil conditioners.

As shown in Figure 2, the Chao1 index, a measure of bacterial community richness, was significantly increased by treatments T1, T2, and T4 compared to the control (CK), with increases of 6.4, 11.1, and 8.9%, respectively (*p <* 0.05). In contrast, no significant differences were observed among treatments for the Shannon and Simpson indices (*p <* 0.05), indicating that bacterial community evenness and overall diversity were not affected by the amendments.

**Figure 3:**
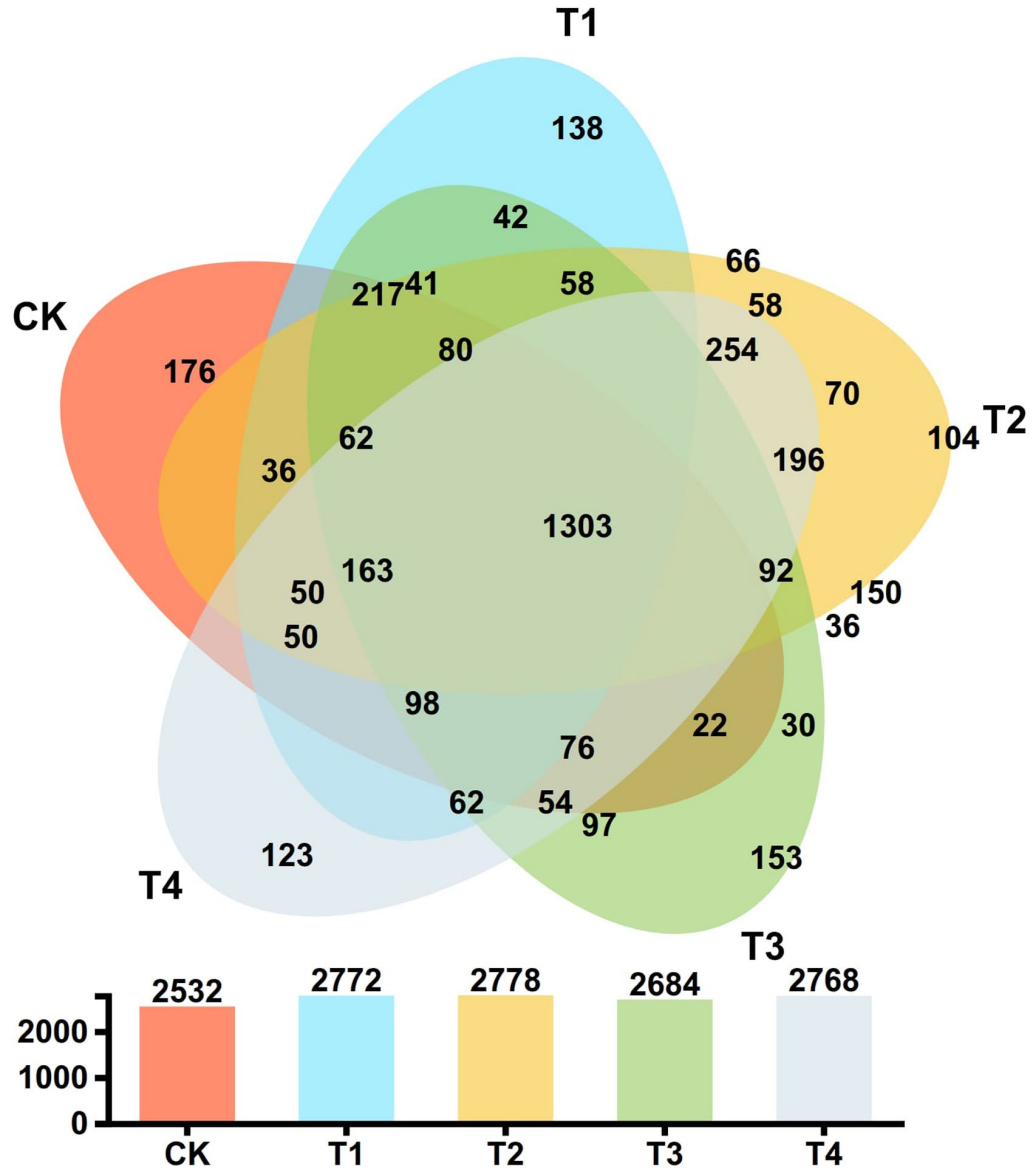
Venn diagrams under different soil conditioners.

A total of 1,303 bacterial species were identified across all five treatments. As illustrated in Figure 3, species abundance followed the order T2 > T4 > T1 > T3 > CK, demonstrating that all soil amendments increased bacterial abundance relative to the unamended control.

### 3.3 Effects of different amendments on bacterial communities at the phylum and genus levels

**Figure 4:**
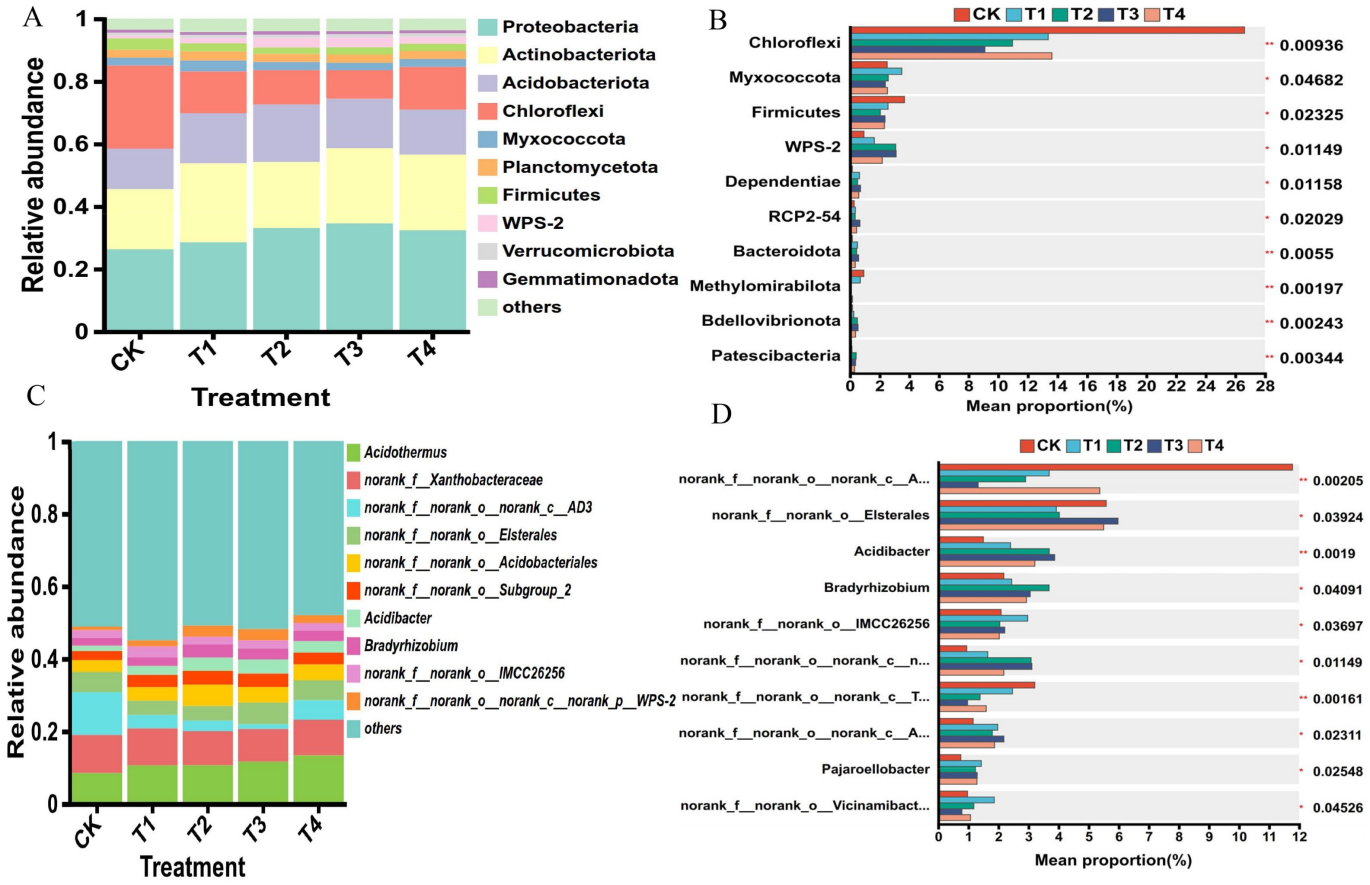
Variation in relative abundance of bacterial phyla under different soil conditioners **(A)**, multi-group comparison analysis at the phylum level **(B)**, relative abundance at the genus level **(C)**, and multi-group comparison analysis at the genus level **(D)**.

At the phylum level, the relative abundances of major bacterial taxa varied among amendment treatments (Figure 4). Compared with the control (CK), the relative abundance of Proteobacteria increased by 25.5% under T2 and by 31.5% under T3. Actinobacteriota increased by 31.8, 25.0, and 26.0% under T1, T3, and T4, respectively. Acidobacteria showed increases of 24.0% (T1), 42.6% (T2), and 22.5% (T3). Myxococcota increased by 40.0% under T1 relative to CK. Notably, the phylum WPS-2 exhibited substantial increases across all amended treatments, with relative abundances rising by 77.8% (T1), 244.4% (T2), 244.4% (T3), and 144.4% (T4) compared with CK.

At the genus level (Figure 4), the relative abundance of *Acidothermus* increased by 26.2% (T1), 26.2% (T2), 38.1% (T3), and 58.3% (T4) relative to CK. *Acidibacter* abundance increased by 60.0% (T1), 146.7% (T2), 160.0% (T3), and 113.3% (T4). For *Bradyrhizobium*, significant increases of 68.2 and 40.9% were observed under T2 and T3, respectively, compared with CK.

Statistical testing revealed that the relative abundances of dominant bacterial phyla and genera differed significantly among soil conditioner treatments. These differences were significant at both the phylum and genus levels (*p <* 0.05), with some taxa showing highly significant variation (*p <* 0.01).

### 3.4 Analysis of soil bacterial beta diversity

**Figure 5:**
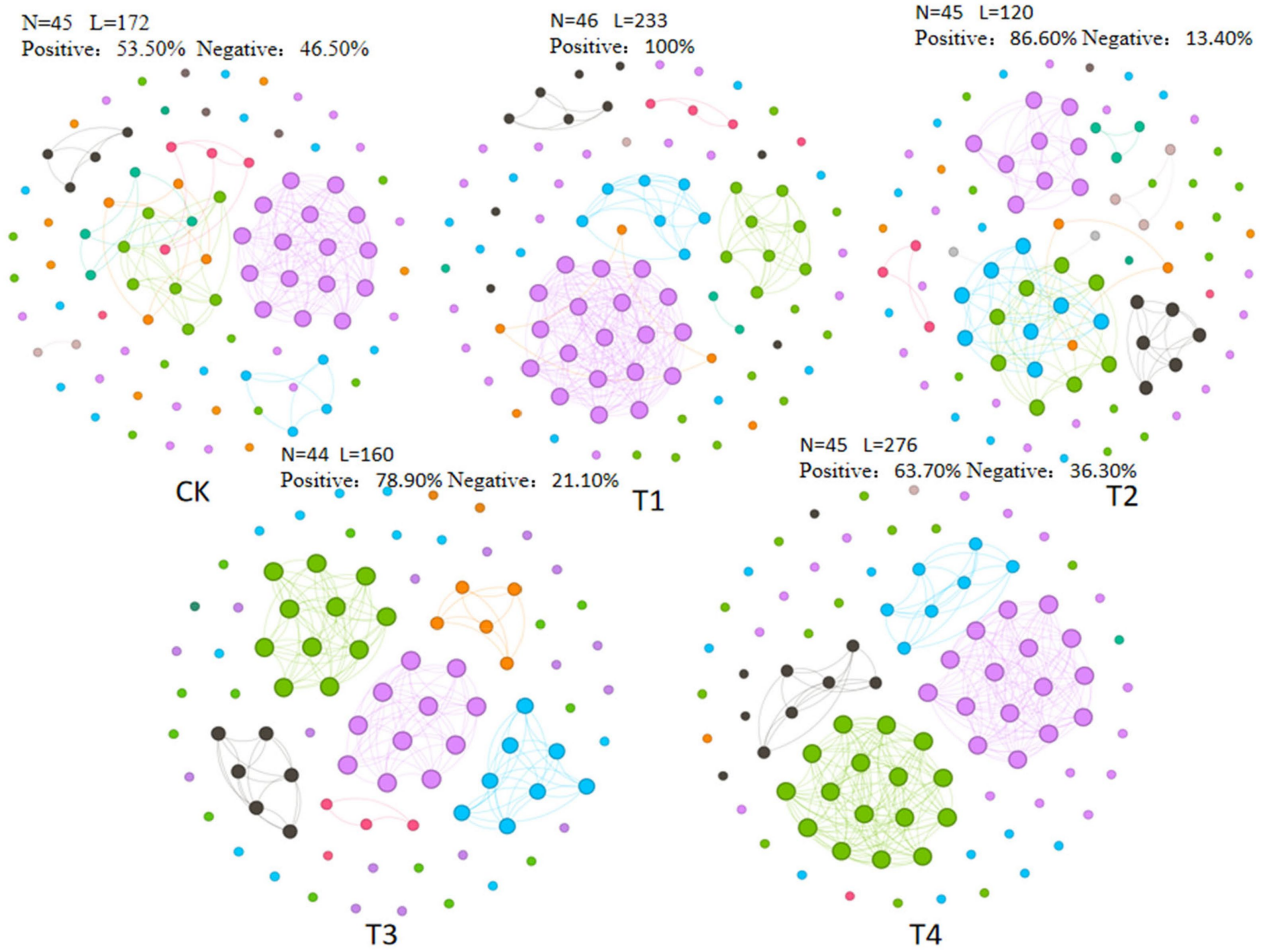
Bacterial co-occurrence network under different soil conditioners.

Principal coordinate analysis (PCoA) revealed distinct clustering of bacterial communities among the different amendment treatments, with all treated samples showing clear separation from the control (CK) (Figure 5). The structure of the bacterial community was significantly altered by the application of soil amendments.

### 3.5 Bacterical ommunity co-occurrence networks

**Figure 6:**
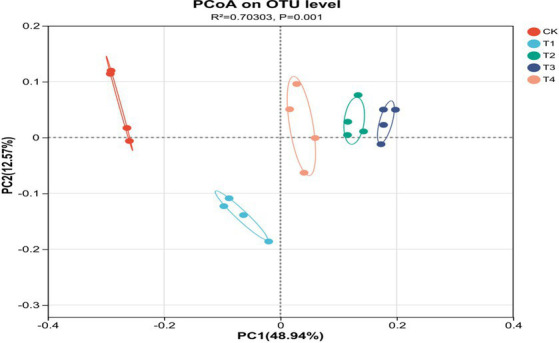
Principal component analysis of bacterial communities under different soil conditioners.

The complexity of bacterial co-occurrence networks in rhizosphere soil was analyzed to evaluate the effects of different amendments on interspecific interactions. Compared with the control (CK), treatments T4 (bio-organic fertilizer combined with polyacrylamide) exhibited higher total edge counts, modularity, and average degree in its network (Figure 6; Table 1), indicating that it enhanced the network complexity of rhizosphere bacterial communities.

**Table 1:** Total network node attributes of bacteria under different soil conditioners.

Treatment	Nodes	Edges	Average degree	Modularity
CK	45	172	3.822	0.492
T1	46	233	5.065	0.438
T2	45	120	2.667	0.783
T3	44	160	3.773	0.74
T4	45	276	6.133	0.613

Nodes, number of operational taxonomic units (OTUs) involved in the co-occurrence network; Edges, number of significant Spearman correlations between OTUs; Average degree, average number of connections per node in the network; Modularity, degree to which the network is divided into independent functional modules.

### 3.6 Relationships between soil bacterial communities, diversity, and physicochemical properties

**Figure 7:**
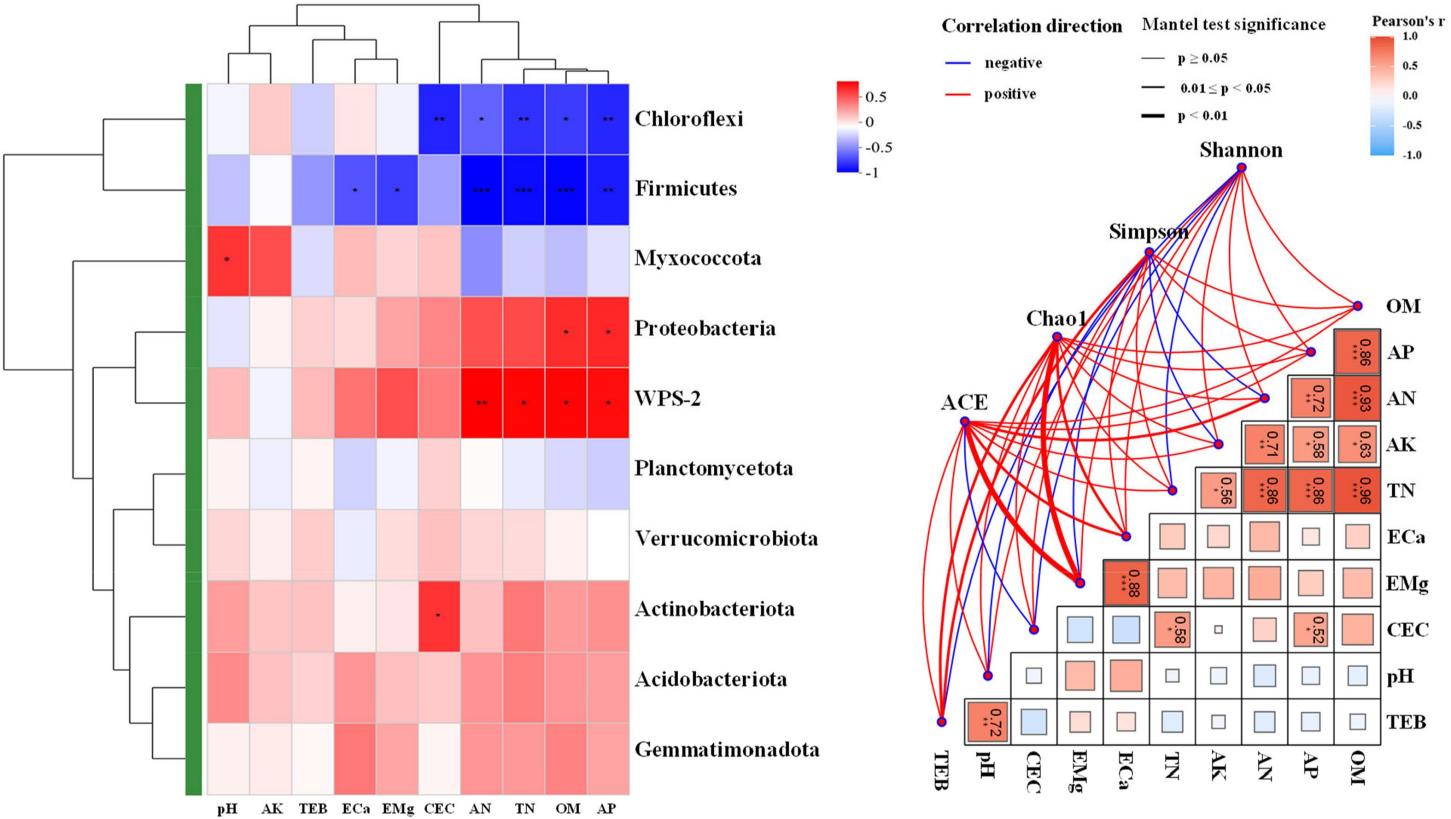
Relationships between soil bacterial communities, diversity, and soil physicochemical properties. Abbreviations: AK, available potassium; AN, available nitrogen; AP, available phosphorus; CEC, cation exchange capacity; ECa, exchangeable calcium, EMg, exchangeable magnesium, OM, organic matter; TN, total nitrogen; TEB, total exchangeable base.

As shown in Figure 7, the correlation heatmap revealed significant associations between specific bacterial phyla and soil chemical properties. Myxococcota exhibited a significant positive correlation with pH and total exchangeable bases (*p <* 0.05). Proteobacteria showed significant positive correlations with TN, AP, and SOM (*p <* 0.05). WPS-2 was significantly positively correlated with AP, organic matter, TN, and exchangeable Mg (*p <* 0.05), and showed an extremely significant positive correlation with AN (*p <* 0.01). Additionally, Actinobacteriota displayed a significant positive correlation with cation exchange capacity (*p <* 0.05).

Mantel analysis revealed significant positive correlations between SOM and multiple soil nutrient indicators, including AP, AN, AK, and TN (*p <* 0.05), indicating that increases in SOM were accompanied by enhanced nutrient availability. Furthermore, bacterial diversity indices showed significant positive correlations with SOM, AP, AN, and TN (*p <* 0.05), suggesting that these environmental factors collectively exert positive effects on bacterial diversity.

**Figure 8:**
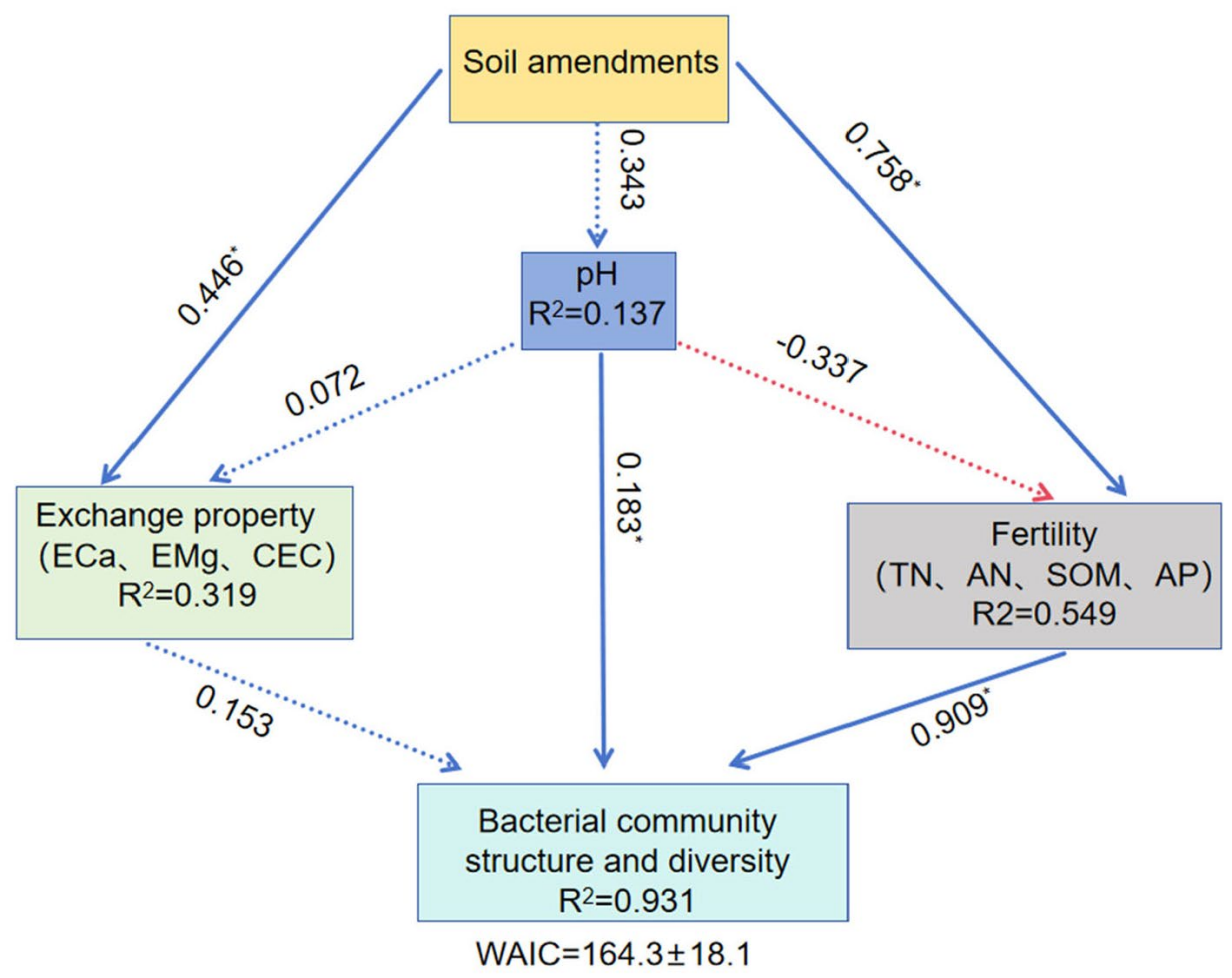
Structural equation models of soil exchange capacity, soil fertility, pH, and bacterial community structure and diversity. The numbers on the arrows are standardized path coefficients. Blue solid arrows and red dashed arrow represent significant positive and negative pathways, respectively, while the blue dashed arrow indicates a non-significant paths. Significance levels are indicated by asterisks: *p < 0.05.

Structural equation modeling (SEM) was used to quantitatively analyze the causal pathways between various factors. The results showed that soil amendments altered bacterial community structure and diversity mainly by affecting soil fertility (path coefficient = 0.909, *p <* 0.05) and pH (path coefficient = 0.183, *p <* 0.05), with an overall explanatory power of 93.10%. Among these, soil fertility was the most important driving factor, whereas the direct effect of pH was relatively weak (Figure 8).

## 4 Discussion

### 4.1 Effects of different amendments on soil physicochemical properties

The application of organic amendments significantly enhanced soil nutrient content in the rubber plantation. In this study, both organic fertilizer and biochar-based treatments (T2 and T3) markedly increased SOM, AP, AN, and TN compared with the unamended control. These improvements can be attributed to the inherent properties of organic fertilizers, which are rich in N, P, K, and organic matter. Their application typically increases soil pH and stimulates microbial activity, leading to improved soil structure, aeration, and permeability. These changes accelerate the decomposition of organic residues and promote nutrient cycling, thereby elevating soil organic matter content (He et al., 2022; Li Z. et al., 2024).

Biochar, characterized by its stable C pools and porous structure with a high specific surface area (Nourmandipour et al., 2021), effectively adsorbs nutrient ions such as N, P, and K. This adsorption reduces nutrient leaching, enhances nutrient availability, and increases the soil’s nutrient retention capacity (Youliang et al., 2023). The beneficial effects of organic amendments on soil fertility are well documented. For instance, long-term fertilization trials have demonstrated that organic fertilizer application significantly increases SOC and TN (Mao et al., 2025). Similarly, biochar application has been shown to elevate SOM, improve soil physicochemical properties, and ameliorate soil acidity (Liu et al., 2023), findings that are consistent with the results of the present study.

Furthermore, in this experiment, organic fertilizer treatments significantly increased the cation exchange capacity (CEC), a key indicator of a soil’s ability to retain and supply nutrients and to buffer against chemical changes. A higher CEC reflects an enhanced capacity to resist acidification by buffering H^+^ and Al^3+^ ions, thereby protecting nutrient ions from leaching and providing a more stable, nutrient-rich physicochemical environment for microbial communities. The humus formed from the decomposition of organic fertilizers is rich in dissociable functional groups, including carboxyl (-COOH) and hydroxyl (-OH). Upon dissociation, these functional groups generate negatively charged sites, thereby enabling soil colloids to adsorb greater quantities of positively charged cationic nutrients (Shi et al., 2019).

### 4.2 Effects of different amendments on bacterial community diversity and structure in rubber plantation soils

Soil ecosystem stability is closely linked to microbial community diversity, with changes in bacterial populations and abundance serving as direct indicators of shifts in the soil environment (Joanna et al., 2023). In this study, the Chao1 index was significantly higher under the T2 treatment (biochar-based fertilizer) than under all other treatments, indicating that biochar application substantially enhanced bacterial species richness. This finding aligns with Sun M. et al. (2025), who reported that biochar application increases bacterial abundance and diversity in soil. The porous structure and large specific surface area of biochar improve the soil microenvironment, creating favorable conditions for bacterial growth and thereby promoting species richness (Jin et al., 2024). However, no significant changes were observed in the Shannon or Simpson indices in this study, suggesting that biochar enhanced species richness without markedly altering community evenness. These results are consistent with previous findings demonstrating that biochar application increases bacterial species richness without affecting community evenness (Jin et al., 2024). The Chao1 index only reflects the species richness of the community (i.e., the number of OTUs) and is extremely sensitive to the increase or decrease of rare species; whereas the Shannon index comprehensively considers species richness and evenness, and is more dependent on the relative abundance distribution of dominant species. The porous structure of biochar provided microhabitats and refuges for a large number of rare species that were originally difficult to survive in acidic and barren soil, significantly increasing the species reserve of the community. However, the absolute abundance of these rare species was still very low and did not change the relative proportion of the original dominant groups, so the community evenness remained stable and the Shannon index did not change significantly (Jin et al., 2024). Notably, some studies have reported a reduction in bacterial diversity following biochar application (Tang, 2025), a finding that contrasts with the present study. This discrepancy suggests that soil type, vegetation type, and management practices modulate the effects of biochar on microbial communities. Future multi-site, long-term trials are needed to further elucidate the underlying mechanisms (Fan et al., 2025; Gu et al., 2025).

Mantel analysis further revealed that bacterial diversity was significantly positively correlated with SOM, available P, and total N (*p <* 0.05), indicating that enhanced nutrient availability is a key driver of increased species richness. Organic C not only provides an energy source for microorganisms but also, through its complex structure, creates additional ecological niches, supporting the coexistence of diverse microbial taxa. Similarly, an adequate supply of available nutrients such as N and P alleviates growth constraints on microorganisms and promotes community proliferation (Noah and Jackson, 2006; Zhang et al., 2022).

Soil microbial community composition is widely recognized as a crucial indicator of soil health. Principal coordinate analysis (PCoA) revealed that the application of different soil amendments significantly altered bacterial community structure (*p <* 0.05). At the phylum level, the dominant bacterial phyla across treatments were Proteobacteria, Actinobacteria, Acidobacteria, Verrucomicrobia, and Myxobacteria, although their relative abundances differed significantly among treatments.

Proteobacteria is recognized as a copiotrophic phylum that plays a key role in soil C and N cycling. The application of organic fertilizers increases SOM content, thereby providing an abundant nutrient supply that promotes the proliferation of copiotrophic bacterial groups. Consistent with this, previous studies have demonstrated that organic fertilizer application enhances the abundance of copiotrophic bacteria such as Proteobacteria in soil (Meiling et al., 2021; Linnan et al., 2020), a finding that aligns well with the results of the present study.

Actinobacteria secrete a range of potent extracellular enzymes, including cellulase, lignin peroxidase, and chitinase, which are specifically adapted to degrade complex organic compounds. Organic fertilizers, such as straw compost and livestock manure, contain substantial amounts of recalcitrant organic matter—including cellulose, hemicellulose, lignin, and chitin—thereby providing suitable substrates for actinobacterial growth and activity. Their application not only supplies these complex organic substrates but also improves soil environmental conditions such as pH and aeration, contributing to the increased relative abundance of Actinobacteria (Zheng et al., 2018).

In contrast, Acidobacteria are generally considered an oligotrophic phylum, primarily involved in the decomposition of recalcitrant compounds such as cellulose and chitin. Their relatively slow C turnover rate is thought to enhance C sequestration in soil (Zheng et al., 2018; Zhuang et al., 2021). Although Acidobacteria are widely considered to prefer nutrient-poor soils (Liu et al., 2024), both organic fertilizer and biochar treatments in this study increased their relative abundance. This finding is consistent with previous research: Organic fertilization has been shown to increase the relative abundance of Acidobacteria in rubber plantation soils (Zhao et al., 2026; Jin et al., 2025) This apparent discrepancy may be attributed to the exceptionally high metabolic diversity within the Acidobacteria phylum (Qiang, 2024). The traditional view holds that Acidobacteria are oligotrophic groups that prefer low-nutrient environments, but the significant increase in the relative abundance of Acidobacteria under organic fertilization in this study is consistent with the results of many recent studies (Dong et al., 2026). The reason is that certain subgroups within the Acidobacteria phylum with some subgroups can decompose complex organic matter such as lignin and humus. The application of organic amendments profoundly changed the soil organic carbon forms: both available carbon and complex carbon increased significantly. The increase in complex carbon provided a new niche for Acidobacteria subgroups with decomposition ability, enabling them to coexist with ‘available carbon specialist’ bacterial groups such as Proteobacteria in eutrophic environments through resource differentiation, rather than being directly excluded by competition (Liu et al., 2024).

In contrast, the relative abundance of Chloroflexi was higher under the control (CK) treatment than under soil conditioner application. This may be explained by the enhanced abundance of Actinobacteria in amended soils, which likely consume more resources. Given the resource competition between Actinobacteria and other phyla, this competitive advantage may restrict the growth and reproduction of other microbial groups. Furthermore, the increase in the relative abundance of Actinobacteria may affect the growth of other groups through resource competition or the secretion of secondary metabolites (such as antibiotics and siderophores) (Wang et al., 2024).

WPS-2 is a newly discovered candidate bacterial phylum, widely distributed in acidic, oligotrophic or stressful environments, with potential phototrophic ability and extreme environmental adaptability (Ward et al., 2019). In this study, the relative abundance of the WPS-2 phylum increased by 244.4% under T2 and T3 treatments compared with CK. The possible reason is that tobacco ash and oil cake are rich in organic matter, and release small molecular organic matter such as amino acids, sugars and vitamins during decomposition. Microorganisms such as WPS-2 may have efficient oligotrophic metabolic capabilities and can preferentially utilize these low-concentration nutrients, thus gaining an advantage in microbial community competition. By optimizing the microenvironment and regulating pH levels, biochar may have provided more favorable conditions for WPS-2 to thrive, thereby promoting its enrichment (Sheremet et al., 2020).

At the genus level, both the biochar (T2) and organic fertilizer (T3) treatments significantly increased the relative abundance of *Bradyrhizobium*. This genus comprises heterotrophic facultative symbionts whose metabolic processes require substantial amounts of organic C. Organic fertilizer provides a high-quality, readily degradable C source, while biochar functions as a stable C reservoir that releases soluble organic C over extended periods. Together, they supply abundant and sustained C and energy sources for these copiotrophic microorganisms (Yu et al., 2025). Concurrently, the N, P, K, and various trace elements released from the amendments provide comprehensive nutritional support for the growth of *Bradyrhizobium* and its host plants (Liang et al., 2022). Thus, biochar and organic fertilizer not only enhance bacterial abundance but also optimize community functional structure through a “C–N–microbe” synergistic effect, thereby establishing a microbiological foundation for the sustainable maintenance of soil health in rubber plantations.

### 4.3 Effects of different amendments on the construction of bacterial networks in rubber plantation soils

Soil microbial networks are crucial to soil health, as greater network complexity is generally associated with higher community stability (Wagg et al., 2019). In this study, the T4 treatment (bio-organic fertilizer + polyacrylamide) exhibited higher total edge counts, modularity, and average degree, indicating that its bacterial network structure was the most complex. This can be attributed to the combined effects of polyacrylamide (PAM) and bio-organic fertilizer. Polyacrylamide improves soil structural stability and water use efficiency while reducing nutrient loss (Parhizkar et al., 2026), thereby influencing microbial community structure (Zhang et al., 2024). This finding is consistent with previous studies demonstrating that polyacrylamide application enhances soil physicochemical properties and microbial habitats (Kay-Shoemake et al., 1998). Concurrently, bio-organic fertilizers are rich in organic matter, humus, and beneficial microorganisms (Bardgett and van der Putten, 2014), providing a continuous supply of nutrients that promote bacterial growth and interspecies interactions, thereby enhancing the connectivity and stability of microbial networks (Li K. et al., 2024). Previous studies have also demonstrated that bio-organic fertilizer application results in a more complex bacterial network structure than inorganic fertilizer (Zhenmin et al., 2022), which aligns with the findings of the present study. Currently, bio-organic fertilizers and polyacrylamide are primarily used individually for soil improvement, with few reports on their combined application. As a linear polymer, polyacrylamide (PAM) can form hydrogen bonds and cationic bridges with humic acids, polysaccharides in bio-organic fertilizers and soil mineral surfaces through its amide groups, thereby promoting the formation and stability of soil microaggregates (Yao et al., 2017). Simultaneously, PAM can improve soil water and fertilizer retention capacity, reduce nutrient leaching, and provide a more stable environment for microbial growth. Therefore, future research should further elucidate the mechanisms by which their combined application influences bacterial network formation.

### 4.4 Analysis of correlations between bacterial community structure and environmental factors

The relationships between microbial community composition and environmental factors were examined in this study. Bacterial community composition exhibited significant positive correlations with SOM, AP, TN, and AN. Structural equation modeling indicated that soil amendments altered bacterial community structure and diversity mainly by affecting soil fertility and pH. As shown in Figure 1, the application of biochar and organic fertilizer significantly enhanced these soil properties. The increase in SOC provided a rich C source for copiotrophic bacteria, thereby promoting the proliferation of taxa capable of decomposing complex organic compounds and consequently enriching bacterial community composition. Similarly, the increase in soil TN altered the N environment, providing an enriched N source for bacteria with high N utilization efficiency while concurrently suppressing bacteria with lower N requirements, thereby driving shifts in bacterial community structure (Dong et al., 2025). In this study, soil fertility was the most important driving factor, and the direct effect of pH was relatively weak. Mainly due to: the spatial variation range of pH value was small, the pH value range of each treatment was 4.12–4.35, and the coefficient of variation was only 2.2%. Moreover, soil nutrient indicators exhibited greater controllability and variability. In this study, the coefficients of variation of organic matter, total nitrogen and available phosphorus were 22.34, 17.43 and 12.30%, respectively.

Previous studies have identified organic matter and TN as primary drivers of bacterial community variation, which is consistent with the findings of the present study (Jinyao et al., 2022; Lan et al., 2023). The results further confirm that SOM, TN, AP, and AN are the main factors driving changes in bacterial community structure. In summary, the “nutrient–acidity” gradient constituted by SOC, AP, TN, and AN represents the primary environmental axis driving the restructuring of bacterial communities in rubber plantation soils.

### 4.5 Outlook

This study systematically elucidated the regulatory effects of different organic amendments on bacterial community structure and co-occurrence networks in the surface soil of acidic rubber plantations in Xishuangbanna, and identified soil fertility as the core factor driving community variation, providing an important theoretical basis for microbial regulation of tropical acidic soils. Although this study focused on medium- to short-term effects and surface soil responses, and did not involve deep soil processes, direct verification of functional genes, and evaluation of production benefit transformation, the results provide a solid foundation for subsequent work. Future studies will build upon these findings to conduct long-term monitoring, further analyze the regulatory mechanisms of amendments on whole-soil-profile microbial communities and carbon and nitrogen cycling functions, optimize precise application schemes and carry out multi-regional joint verification, and simultaneously evaluate their effects on rubber tree growth and long-term ecological risks, thereby providing more comprehensive scientific support for the sustainable management of rubber plantation soils.

## 5 Conclusion

The application of soil amendments significantly enhanced soil fertility and buffering capacity, with tobacco-based organic fertilizer and biochar-based fertilizer demonstrating the most pronounced ameliorative effects. Analysis of bacterial community structure revealed that treatments with organic fertilizer and biochar significantly increased the relative abundance of key functional phyla, including *Proteobacteria* and *Acidobacteria*. Moreover, organic matter, total nitrogen, available phosphorus, and alkali-hydrolyzable nitrogen were identified as the primary environmental factors driving variation in community structure. Structural equation modeling indicated that soil conditioners alter bacterial community structure and diversity by influencing soil fertility. In summary, the combined application of high-quality organic fertilizer and biochar confers three principal benefits: enhanced soil fertility, acidic soil pH stabilization, and promotion of microbial activity. This integrated approach achieves the dual regulatory objectives of “chemical improvement and microbial restoration” in soil, providing a theoretical foundation and technical support for the sustainable management and green production of acidified rubber plantation soils. As the experimental period of this study was limited to 2 years, the long-term effects of these soil amendments on soil quality warrant further verification through extended field trials.

## Data Availability

The original contributions presented in the study are publicly available. This data can be found at: http://www.ncbi.nlm.nih.gov/bioproject/1481468, PRJNA1481468.
